# Enhanced microalgal lipid production with media engineering of potassium nitrate as a nitrogen source

**DOI:** 10.1080/21655979.2017.1316440

**Published:** 2017-05-04

**Authors:** Rakesh Singh Gour, Madhusudan Bairagi, Vijay Kumar Garlapati, Anil Kant

**Affiliations:** Department of Biotechnology and Bioinformatics, Jaypee University of Information Technology, Waknaghat, HP, India

**Keywords:** *Chlorella* sp, growth kinetics, lipid productivity, potassium nitrate, *Scenedesmus* sp.

## Abstract

Algal biofuels are far from a commercial reality due to the technical challenges associated with their growth and lipid extraction procedures. In this study, we investigated the effect of 4 different media and 5 different nitrogen sources at 5 levels on the growth, biomass and lipid productivity of *Scenedesmus* sp and *Chlorella* sp The hypothesis was that a nitrogen source can be identified that provides enough stress to accumulate lipids without compromising significantly on biomass and lipid productivity. A maximum specific growth rate and doubling per day have been observed with algal species using modified BG-11 medium. Among the tested nitrogen sources, 2.5 mM potassium nitrate as a nitrogen constituent of modified BG-11 medium resulted in higher lipid content and productivity in the case of *S. dimorphus* (29.15%, 15.449 mg L^−1^day^−1^). Another noteworthy outcome of the present study lies in the usage of a smaller amount of the nitrogen source, i.e., 2.5 mM, which is found to be 7 times less than the standard BG11 media (17.60 mM sodium nitrate).

## Introduction

Recently, microalgae have been receiving renewed attention from the research community as potential sources of biodiesel feedstock as well as of various industrial commodities. Feedstock scarcity and pretreatment technical problems of other non-edible oils can be alleviated through the usage of fast growing, high yield algal species. The technical feasibility of growing algae for biodiesel production through microalgal lipid transesterification has been well acknowledged in the scientific literature.^1-3^ Many government and private firms are actively engaged in algal research for biodiesel production.^4^ Despite immense potential and concerted research efforts, algal biofuels are far from a commercial reality due to the technical challenges associated with the upstream, downstream and scaling aspects of the microalgal processes. The key upstream challenges are species/strain selection, optimization of bioprocesses and genetic improvement of the selected species/strains. Isolation of algal species from indigenous places and determining their suitability for biofuel production are the predominant steps.^5-11^ Our previous studies reported the indigenous algal species of Himachal Pradesh, India, which include *Scenedesmus dimorphus, Scenedesmus quadricauda* and *Chlorella* sp Based on the lipid content, biomass and lipid productivity profiles, we have selected these particular isolates for our ongoing research activities.^12,13^

The microalgal isolates were evaluated under normal growth conditions (nutrient sufficient) to reveal their genetic potential to produce and accumulate lipids. The lipid content and productivity of the selected microalgae isolates can be further increased by optimizing growth-determining factors. An ideal process should be able to produce microalgae with high lipid content at the highest productivity. Attention must be paid to optimizing the cultivation practices leading to high biomass and lipid productivity. One of the most important factors which directly affects the production of high biomass and the lipid content of algae is media composition.^14,15^ Various media with different compositions have been developed for isolation and cultivation of microalgae and can be tested for the cultivation of microalgae species.

Among the media components, nitrogen is an essential nutrient affecting the biomass growth and lipid productivity of various microalgae.^14^ Research so far on different microalgae species suggests that higher oil accumulation is attained under nutrient starvation conditions, especially nitrogen starvation, but much lower biomass growth is attained under such conditions, which results in low overall lipid productivity.^15,16^ In light of this, the selection of a suitable growth medium and optimization of the nitrogen source for high biomass and lipid productivity of potential strains of microalgae seems to be the most obvious starting point to improve the biomass and lipid productivity. The hypothesis is that a nitrogen source can be identified that provides enough stress to accumulate lipids without comprising significantly on biomass and lipid productivity.

In this study, we investigated the effect of 4 different media and, subsequently, 5 different nitrogen sources at 5 levels on the growth, biomass and lipid productivity of the best-selected isolates of 3 microalgae viz. *Scenedesmus dimorphus*, *Scenedesmus quadricauda* and *Chlorella* sp.

## Results and discussion

### Selection of suitable medium for growth and lipid production

The data regarding the effects of different growth media on the biomass and lipid accumulation in 3 different microalgae is presented in Table 1. The highest biomass and total lipids, irrespective of the microalgae species, were recorded when microalgae are grown in modified BG-11 medium, followed by Chu-10. The F/2 medium was found to be least productive for the studied parameters in all 3 isolates. On the other hand, *Scenedesmus dimorphus* performed better than the other 2 species on all media tested, which once again proves its superiority.^13^ Selection of an optimal culture medium will be a key factor in exploiting the potential of microalgae for lipid and bioactive compound production. Given the diverse nature of microalgae species, no single medium would be suitable for higher growth and production in all the species, so it is worth trying a few different formulations that are frequently used and found in the literature. In recent years, there have been several scientific reports describing the selection and standardization of optimum media for growth, biomass accumulation and specific cultivation objectives of different microalgae.^17-23^ The results are in accordance with Sharma et al. 2016, who reported that modified BG-11 was the most economical and efficient medium for Chlorella sp among the tested media.^23^ Similarly. Rios et al. 2015 reported the superior results of BG-11 medium over Guillard f/2 media on the growth and lipid accumulation of *Desmodesmus* sp.^21^

**Table 1.** Effect of different growth media on total biomass and Lipid content of microalgae isolates (Each value is Mean ± SD (n = 3).

Media	BG-11	CHU-10	BBM	F/2
*Scenedesmus quadricauda* (*Sq19*)				
Biomass g L^−1^	0.715 ± .035	0.481 ± .010	0.306 ± .005	0.145 ± .004
Lipid %	13.12 ± .020	11.13 ± .015	9.11 ± .020	7.91 ± .015

*Scenedesmus dimorphus* (*Sd12*)				
Biomass g L^−1^	0.753 ± .006	0.623 ± .003	0.545 ± .002	0.416 ± .003
Lipid %	27.16 ± .032	23.27 ± .026	19.96 ± .020	15.14 ± .041

*Chlorella* species (*Chl.16*)				
Biomass g L^−1^	0.490 ± .006	0.307 ± .005	0.282 ± .002	0.201 ± .004
Lipid %	24.97 ± .041	19.08 ± .043	15.16 ± .030	13.93 ± .030

The growth of algal cultures is affected by the macro and micronutrient composition in the media and their availability to cultures. Many media do not contain an optimum balance of macro and micronutrients. Macronutrients such as nitrogen, potassium, magnesium, sulfur, and sodium are non-toxic to algal cells and so can be added at higher concentrations. In contrast, essential micronutrients such as Fe, Cu, Mn, Zn, Co and Mo are growth-limiting at low concentrations and toxic at higher concentrations.^24,25^ These micronutrients play a critical role in many metabolic pathways that influence the growth of algal cultures. The chemical composition of 4 media tested in this study is shown in Table 2. A close perusal of Table 2 indicates that modified BG-11 medium has a fair balance of micronutrients regarding both their presence and concentration. Many of these micronutrients are lacking in CHU-10 medium, while their concentration in BBM and F/2 media is much higher than in modified BG-11. Therefore, the optimum balance of micronutrients in the modified BG-11 medium for the investigated algae species could be one of the reasons for higher growth in this medium. Modified BG-11 medium also contains citric acid as an additional component and iron (Fe) in form of ferric ammonium citrate. Citric acid solubilizes the salts, preventing precipitation and thus increasing their availability to cells and likely resulting in a higher growth rate.^26^

**Table 2.** Chemical composition of 4 different culture media used.^17-20,47^

Concentration (g L^−1^)	BG-11	CHU-10	BBM	F/2
Na_2_MG EDTA	1.00	—	—	—
Ferric ammonium citrate	6.00	—	—	—
Citric Acid · H_2_O	6.00	—	—	—
CaCl_2_ · 2H_2_O	27.0	—	02.50	—
MgSo4 · 7 H2O	75.0	12.50	07.50	—
K_2_HPO_3_ · 3 H2O	40.0	02.50	07.50	—
(OR K_2_HPO_4_)	39.0	—	17.50	—
H_3_BO_3_	2.86	02.48	11.42	—
MnCl_2_ · 4 H_2_O	—	—	01.44	180.0
ZnSO_4_ · 7 H_2_O	0.22	0.23	08.88	22.0
CuSO_4_ · 5 H_2_O	79.0	0.10	01.57	09.80
NaMoSO_4_ · 2 H_2_O	21.0	—	0.71	06.30
NaNO_3_	1.500	—	25.00	75.0
Na_2_CO_3_	20.0	10.0	—	—
Co(NO_3_)2 · 6H_2_O	—	0.14	0.49	—
NaCl	—	02.50	2.50	—
NaHCO_3_	—	—	—	—
Na_2_SiO_3_ · 9H_2_O	—	12.50	—	30.0
EDTA & KOH	—	—	50 and 31	04.36
Thiamine (B_1_)	—	0.05 g	—	0.20
Biotin	—	2.50 g	—	01.0
vitamin B_12_	—	2.50 g	—	01.0
EDTA	—	—	—	—
MoO_3_	—	—	0.71	—
NaH_2_PO_4_ · H_2_O	—	—	—	05.0
FeCl_3_ · 6H_2_O	—	—	—	3.15
CoCl_2_ · 6H_2_O	—	—	—	10.0

### Effect of nitrogen sources on growth kinetics

**Figure 1.**
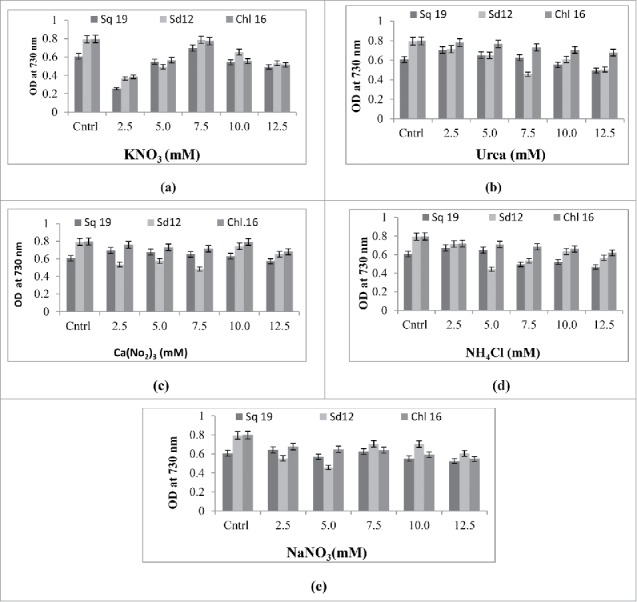
Growth responses of *S. quadricauda (*sq *19), S. dimorphus* (sd *12,*) and *Chlorella sp* (Chl 16) isolates (after 18th day) at OD at 720 nm in modified BG-11 medium supplemented with (A) KNO_3_ (B) Urea (C) Ca(NO_3_)_2_ (D) NH_4_Cl (E) NaNO_3_· All values are represented as ± s.d of 3 replications.

**Figure 2.**
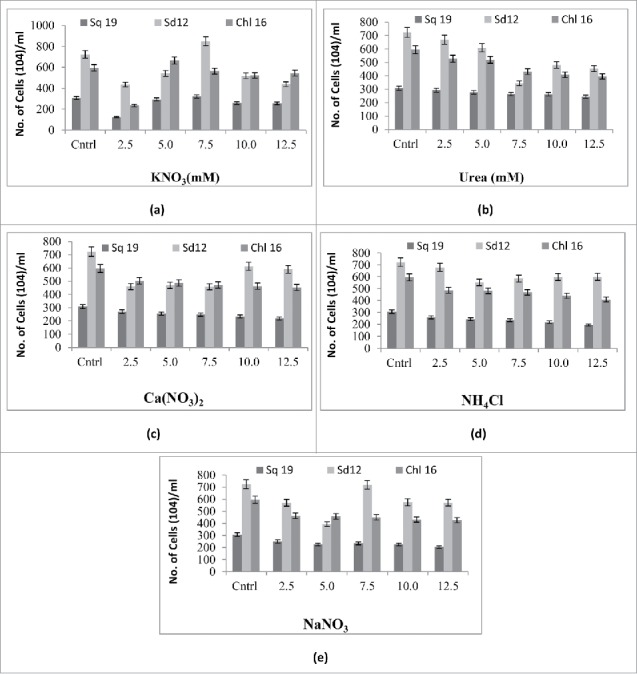
Growth responses of *S. quadricauda (*sq *19), S. dimorphus* (sd *12,*) and *Chlorella sp* (Chl 16) isolates (after 18th day) measured as cell density/ml in modified BG-11 medium supplemented with (A) KNO_3_ (B) Urea (C) Ca(NO_3_)_2_ (D) NH_4_Cl (E) NaNO_3_· All values are represented as ± s.d of 3 replications.

The growth response of the 3 algal species, measured as absorbance at 730 nm on the 18th day of culture, and the cell density in modified BG-11 medium supplemented with 5 nitrogen sources at 5 levels along with a control are depicted in Figure 1 and Figure 2, respectively. The control consisted of modified BG-11medium with the usual 17.60 mM NaNO_3_. The absorbances of the algal cultures of *S. quadricauda*, *S. dimorphus* and *Chlorella* sp were 0.6, 0.8 and 0.8, respectively, in the control medium. Upon careful perusal of Figure 1, it is evident that only media supplemented with KNO_3_ at a concentration of 7.5mM per liter could match the control medium in terms of growth measured by the absorbance at 730nm. Similarly, the number of cells per ml of culture were recorded as 300 × 10^4^, 700 × 10^4^ and 600 × 10^4^ in the case of *S. quadricauda*, *S. dimorphus* and *Chlorella* sp in the control medium, and the cell counts were equivalent or better only in the medium supplemented with 7.5 mM of KNO_3_ (Fig. 2). However, the media supplemented with 2.5 mM urea and 12.5 mM Ca(NO_3_)_2_ were also very close to the control medium in terms of both absorbance and cell density of the all 3 microalgae cultures. The trends agree with Arumugam et al. 2013,^16^ who concluded that *Scenedesmus bijugatus* preferred nitrates of sodium and potassium as a nitrogen source. Tables 3–5 shows the growth rate kinetics of microalgae *S. quadricauda*, *S. dimorphus* and *Chlorella* sp in modified BG-11 medium supplemented with 5 nitrogen sources at 5 levels. The effects of all the treatments were statistically significant. The maximum specific growth rate and doubling per day for *S. quadricauda* (0.244, 0.352), *S. dimorphus* (0.297, 0.428) and *Chlorella* sp (0.302, 0.435)were observed when cultured in BG11 medium supplemented with KNO_3_ at concentrations of 7.5 mM, 7.5 mM and 5 mM, respectively. These results once again confirm the superiority of KNO_3_ as nitrogen source over other treatments, including the control BG-11 medium, which contains NaNO_3_ as its nitrogen source at a concentration of 17.6 mM. Therefore, from this data, it is concluded that KNO_3_ at a concentration of 5–7.5 mM in BG-11 medium results in better growth of the microalgae under investigation.

**Table 3.** Summary of Growth kinetics, biomass and lipid production in *Scenedesmus quadricauda Sq19* isolate, in BG-11 medium supplemented with different nitrogen sources.

Nitrogen Concentrations		Specific growth rate (µ)^***^	Doubling per day (K)^***^	Biomass g L^−1^^***^	Biomass productivity (Pdwt) g L^−1^ day^−1^^***^	Lipids content (Lc) (%)^***^	Lipid productivity (Lp) mg L^−1^ day^−1^^***^
(BG-11)	Control	0.152^bc^	0.219^cd^	0.736^p^	0.043^ab^	13.12^efghij^	5.640 g^h^
KNO_3_	2.5 mM	0.136^abc^	0.197^abcd^	0.731^t^	0.057^ab^	17.63^k^	10.049^i^
	5 mM	0.155^c^	0.224^d^	0.845^s^	0.049^ab^	13.41^efghij^	6.571^h^
	7.5 mM	0.244^bc^	0.352^bcd^	0.983^op^	0.040^ab^	11.26^cdefg^	4.509^fg^
	10 mM	0.151^bc^	0.215^cd^	0.709^o^	0.041^ab^	11.75^defghi^	4.817^fg^
	12.5 mM	0.151^bc^	0.219^cd^	0.681^n^	0.040^ab^	11.26^defgh^	4.504^ef^
Urea	2.5 mM	0.144^bc^	0.207^bcd^	0.586^j^	0.034^b^	10.78^bcdef^	3.665^de^
	5 mM	0.147^bc^	0.212^bcd^	0.579^ij^	0.035^ab^	14.90^hijk^	5.215^fg^
	7.5 mM	0.149^bc^	0.212^bcd^	0.727^p^	0.042^ab^	12.84^efghij^	5.3928^fg^
	10 mM	0.149^bc^	0.215^cd^	0.735^pq^	0.043^bc^	10.44^bcde^	4.4892^ef^
	12.5 mM	0.143^bc^	0.206^bcd^	0.704^o^	0.041^ab^	8.993^abcd^	3.687^de^
Ca(NO_3_)_2_	2.5 mM	0.139^abc^	0.199^abcd^	0.411^e^	0.024^a^	11.36^defgh^	2.726^abcd^
	5 mM	0.151^bc^	0.218^cd^	0.283^cd^	0.016^a^	16.46^jk^	2.633^abcd^
	7.5 mM	0.145^bc^	0.209^bcd^	0.205^a^	0.012^a^	14.61^ghijk^	1.753^a^
	10 mM	0.132^abc^	0.191^abcd^	0.495^g^	0.029^ab^	10.77^bcdef^	3.123^bcd^
	12.5 mM	0.136^abc^	0.177^ab^	0.572^i^	0.033^ab^	7.570^abc^	2.498^abc^
NH_4_Cl	2.5 mM	0.135^abc^	0.195^abcd^	0.282^c^	0.016^a^	15.38^ijk^	2.460^abc^
	5 mM	0.135^abc^	0.195^abcd^	0.292^d^	0.017^a^	13.36^efghij^	2.271^abc^
	7.5 mM	0.142^abc^	0.204^bcd^	0.251^b^	0.014^a^	13.27^efghij^	1.857^a^
	10 mM	0.152^bc^	0.196^abcd^	0.257^b^	0.015^a^	14.25^fghijk^	2.137^ab^
	12.5 mM	0.116^a^	0.167^a^	0.771^r^	0.045^ab^	5.613^a^	2.525^abc^
NaNO_3_	2.5 mM	0.145^bc^	0.210^bcd^	0.664^m^	0.039^ab^	8.083^abcd^	3.152^cd^
	5 mM	0.141^abc^	0.203^abcd^	0.644^i^	0.037^ab^	7.250^ab^	2.682^abcd^
	7.5 mM	0.128^ab^	0.184^abc^	0.557^h^	0.032^ab^	6.510^a^	2.083^a^
	10 mM	0.138^abc^	0.200^abcd^	0.631^k^	0.037^ab^	7.397^ab^	2.736^abcd^
	12.5 mM	0.131^abc^	0.189^abcd^	0.454^f^	0.026^a^	7.343^ab^	1.909^a^
Std. Error		0.00128	0.0187	0.02323	0.0417	0.38124	0.2114

* Significant at p < 0.05,

** Significant at p < 0.01,

*** Significant at p < 0.001,

ns Non significant, a–q = Means in the column with same superscript letter are not significantly different as measured by 2 sided Tukey's – bpost-hoc range test between isolates

**Table 4.** Summary of Growth kinetics, biomass and lipid production in *Scenedesmus dimorphus Sd12* isolate, in BG-11 medium supplemented with different nitrogen sources.

Nitrogen Concentrations		Specific growth rate (µ)^***^	Doubling per day (K)^***^	Biomass g L^−1^^***^	Biomass productivity (Pdwt) g L^−1^ day^−1^^***^	Lipids content (Lc) (%)^***^	Lipid productivity (Lp) mg L^−1^ day^−1^^***^
(BG-11)	Control	0.159^l^	0.175^k^	0.751^g^	0.044^bcd^	27.96^i^	12.302^cdefg^
KNO_3_	2.5 mM	0.171^kl^	0.288^jk^	0.800^hi^	0.053^cde^	29.15^hi^	15.449^g^
	5 mM	0.249^a^	0.216^a^	0.932^l^	0.054^cde^	27.89^ghi^	15.061^cg^
	7.5 mM	0.297^defghi^	0.428^defgh^	1.001^n^	0.058^de^	24.97^efgh^	14.482^fg^
	10 mM	0.213^fghij^	0.307^fghij^	0.826^jk^	0.048^cde^	24.21^cdef^	11.621^bcdefg^
	12.5 mM	0.223^hijk^	0.322^ijk^	0.877^k^	0.051^cde^	20.52^abc^	10.465^abcdefg^
Urea	2.5 mM	0.232^jkl^	0.334^ijk^	0.902^o^	0.050^cde^	27.99^ghi^	13.991^efg^
	5 mM	0.216^hijk^	0.018^ghij^	0.839^j^	0.049^cde^	25.02^defg^	12.259^bcdefg^
	7.5 mM	0.182^abcd^	0.263^bcdef^	0.925^l^	0.054^cde^	22.70^cde^	12.258^bcdefg^
	10 mM	0.219^hijk^	0.317^hij^	0.778^gh^	0.045^bcde^	20.56^abcd^	9.252^abcdefg^
	12.5 mM	0.193^cdefgh^	0.279^cdefg^	0.612^de^	0.036^abc^	17.97^a^	6.469^ab^
Ca(NO_3_)_2_	2.5 mM	0.199^defghi^	0.287^defghi^	0.648^ef^	0.038^abcd^	24.69^defg^	9.382^abcdefg^
	5 mM	0.178^abcde^	0.257^abcde^	0.566^c^	0.033^abc^	19.43^abc^	6.411^ab^
	7.5 mM	0.194^cdefgh^	0.280^cdefg^	0.600^cd^	0.035^abc^	18.33^ab^	6.415^abc^
	10 mM	0.164^abc^	0.237^abc^	0.444^b^	0.026^ab^	18.01^a^	4.682^a^
	12.5 mM	0.157^ab^	0.226^ab^	0.601^cd^	0.035^abc^	18.30^a^	6.405^ab^
NH_4_Cl	2.5 mM	0.228^hijk^	0.330^ghij^	0.833^jk^	0.049^cde^	27.61^fghi^	13.529^defg^
	5 mM	0.204^ghij^	0.295^fghij^	0.585^cd^	0.034^abc^	25.64^efghi^	8.717^abcde^
	7.5 mM	0.220^hijk^	0.318^ghij^	0.582^cd^	0.034^abc^	24.05^bcde^	8.177^abcd^
	10 mM	0.182^abcd^	0.262^abcd^	0.601^cd^	0.035^abc^	21.63^cde^	7.570^abcd^
	12.5 mM	0.176^abcdef^	0.254^abcde^	0.345^a^	0.020^a^	20.28^a^	4.056^a^
NaNO_3_	2.5 mM	0.182^abcd^	0.262^abcd^	0.886^l^	0.052^cde^	27.08^ghi^	14.081^efg^
	5 mM	0.186^abcd^	0.268^abcd^	0.950^n^	0.055^cde^	26.31^efgh^	14.470^defg^
	7.5 mM	0.209^efghij^	0.302^efghi^	1.160^o^	0.068^e^	20.68^abc^	14.062^efg^
	10 mM	0.157^ab^	0.227^ab^	0.668^f^	0.039^abcd^	22.45^cdef^	8.755^abcdef^
	12.5 mM	0.166^abc^	0.239^abc^	0.837^ij^	0.049^cde^	17.92^a^	8.781^abcdef^
Std. Error		0.00335	0.00489	0.02303	0.00140	0.40892	0.00043

* Significant at p < 0.05,

** Significant at p < 0.01,

*** Significant at p < 0.001,

ns Non- significant, a- o = Means in the column with same superscript letter are not significantly different as measured by 2 sided Tukey's – bpost-hoc range test between isolates

**Table 5.** Summary of Growth kinetics, biomass and lipid production in *Chlorella* sp *Chl16* isolate, in BG-11 medium supplemented with different nitrogen sources.

Nitrogen Concentrations		Specific growth rate (µ)^ns^	Doubling per day (K)^ns^	Biomass g L^−1^^***^	Biomass productivity (Pdwt) g L^−1^ day^−1^^***^	Lipids content (Lc) (%)^***^	Lipid productivity (Lp) mg L^−1^ day^−1^^***^
(BG-11)	Control	0.108^a^	0.201^a^	0.484^kl^	0.0265^i^	14.65^c^	3.8^fghi^
KNO_3_	2.5 mM	0.294^a^	0.344^a^	0.424^l^	0.0311^m^	28.13^g^	8.7^no^
	5 mM	0.302^a^	0.435^a^	0.523^kl^	0.0308^lm^	22.04^e^	6.8^m^
	7.5 mM	0.296^a^	0.383^a^	0.236^c^	0.0133^c^	19.98^d^	2.7^bc^
	10 mM	0.205^a^	0.296^a^	0.287^d^	0.0165^de^	16.74^c^	2.9^bcde^
	12.5 mM	0.196^a^	0.283^a^	0.353^f^	0.0208^fg^	15.61^c^	3.2^defg^
Urea	2.5 mM	0.151^a^	0.289^a^	0.681^n^	0.0400^o^	23.170^e^	9.3^o^
	5 mM	0.183^a^	0.265^a^	0.112^a^	0.0060^a^	19.940^d^	1.2^a^
	7.5 mM	0.183^a^	0.264^a^	0.159^b^	0.0090^b^	15.770^c^	1.4^a^
	10 mM	0.175^a^	0.254^a^	0.497^kl^	0.0290^kl^	13.280^b^	3.9 g^h^
	12.5 mM	0.194^a^	0.280^a^	0.329^m^	0.0367^n^	11.103^a^	4.1^hi^
Ca(NO_3_)_2_	2.5 mM	0.197^a^	0.275^a^	0.325^m^	0.0367^n^	23.107^e^	8.4^n^
	5 mM	0.197^a^	0.284^a^	0.419^hi^	0.0240 g^h^	19.940^d^	4.8^j^
	7.5 mM	0.195^a^	0.282^a^	0.326^m^	0.0190^g^	13.280^b^	2.5^bc^
	10 mM	0.189^a^	0.273^a^	0.361^fg^	0.0210^fg^	15.770^c^	3.3^efg^
	12.5 mM	0.190^a^	0.273^a^	0.532^lm^	0.0310^lm^	19.940^d^	6.2^kl^
NH_4_Cl	2.5 mM	0.185^a^	0.267^a^	0.268^cd^	0.0150^cd^	19.940^d^	3.0^cdef^
	5 mM	0.198^a^	0.286^a^	0.301^de^	0.0170^e^	13.280^b^	2.3^b^
	7.5 mM	0.186^a^	0.268^a^	0.178^b^	0.0100^b^	15.770^c^	1.6^a^
	10 mM	0.182^a^	0.262^a^	0.452^kl^	0.0260^i^	23.107^e^	6.0^k^
	12.5 mM	0.181^a^	0.261^a^	0.333^ef^	0.0190^f^	15.770^c^	3.0^cdef^
NaNO_3_	2.5 mM	0.198^a^	0.286^a^	0.521^kl^	0.0300^lm^	23.170^e^	7.0^m^
	5 mM	0.190^a^	0.275^a^	0.339^ef^	0.0193^fg^	19.983^d^	3.9 g^h^
	7.5 mM	0.181^a^	0.261^a^	0.304^de^	0.0170^e^	15.770^c^	2.8^bcd^
	10 mM	0.182^a^	0.263^a^	0.485^kl^	0.0280^jk^	15.770^c^	4.4^ij^
	12.5 mM	0.186^a^	0.269^a^	0.395 g^h^	0.0230^h^	15.607^c^	3.6^fgh^
Std. Error		0.00139	0.00202	0.01672	0.00099	0.48021	0.00026

* Significant at p < 0.05,

** Significant at p < 0.01,

*** Significant at p < 0.001,

ns Non- significant, a- o = Means in the column with same superscript letter are not significantly different as measured by 2 sided Tukey's – b post-hoc range test between isolates.

### Effect of nitrogen sources on biomass and lipid production

The biomass yield, biomass productivity, lipid content and lipid productivity measured from the harvested algal biomass obtained from all experimental treatments have also been tabulated in Tables 3–5. The effect of all nitrogen sources and their concentrations was found to be statistically significant at the 0.001 level of significance for the *Scenedesmus* sp studied. In the case of *Scenedesmus quadricauda*, the highest biomass yield (0.983 gL^−1^) and biomass productivity (0.057 gL^−1^day^−l^) were obtained when cultured in modified BG-11 medium with 7.5mM and 2.5mM of KNO_3_, respectively, which were followed by medium supplemented with 5.0 mM KNO_3_ (Table 3). However, the highest lipid content (17.63%) and lipid productivity (10.049mgL^−1^day-^l^) were recorded when nitrogen was supplied as KNO_3_ at a concentration of 2.5mM, which was closely followed by 5 mM KNO_3_. Urea at a concentration of 5.0mM resulted in at par biomass productivity and marginally less lipid content and lipid productivity compared with the best treatment, whereas the rest of the treatments yielded either poor biomass lipid content or poor productivity compared with the control medium.

It is evident from the data depicted in Table 4 that S*. dimorphus* produced its highest biomass yield (1.160 gL^−l^) and productivity (0.068 gL^−1^day^−l^) when cultured in modified BG-11 medium with NaNO_3_ at a concentration of 7.5 mM, which was closely followed by KNO_3_ at 7.5 mM. However, the lipid content and productivity of this species were lower in both these treatments compared with those recorded with modified BG-11 medium supplemented with 2.5mM KNO3(29.15%, 15.449 mgL^−1^day^−l^). Similar trends of results were obtained in the case of *Chlorella* sp, in which higher biomass (0.681 gL^−1^), biomass productivity (0.04 gL^−1^day^−1^), lipid productivity (9.3mgL^−1^day^−l^) and lipid content (28.13%) were obtained using modified BG-11 medium with KNO_3_ as a nitrogen source (Table 5). Another notable combination of nitrogen sources and concentrations which resulted in significant growth and lipid accumulation was the medium with 5.0 mM of both KNO_3_ and NaNO_3_.

High lipid content has been reported in microalgae under nutrient starvation conditions, ignoring the overall lipid productivity.^27-32^ Hakalin et al. 2014 studied the effects of nitrogen, phosphorus and vitamins on the growth and lipid content of a *Scenedesmus* sp and reported a maximum lipid content of 29.3%.^33^ The present results also agree with Arumugam et al. 2013, who found thatnitrate at lower concentrations (5–10mM) was a better source of nitrogen in *Scenedesmus bijugatus.*^16^ Similarly, Pancha et al. 2014 also concluded that removal of nitrate from the growth medium results in higher lipid content (27.93%) in *Scenedesmus* sp CCNM 1077.^34^ It has been suggested that under nitrogen limitation conditions, microalgal cells divert more photosynthetically derived energy to making storage products such as lipids and carbohydrates, but very small initial nitrogen concentrations may also have a deleterious effect, as noted by Sanchez-Garcia et al. 2013 in *Tetraselmis suecica* and *Chlorella minutissima.* The lipid accumulation increased from 8% to 17.5% in *Tetraselmis suecica* and 22.7% to 36.6% in *Chlorella minutissima* when an initial sodium nitrate concentration was increased from 57 to 225 mg L^−l^ and from 57 to 113 mg L^−l^, respectively.^35^ Lipid productivities of 24.66 mg L^−1^ day ^−1^ and 16.11 mg L^−1^ have been reported by Jena et al. 2012^8^ in *Scenedesmus* sp and *Chlorella* sp, respectively. In another study by Rodolfi et al. 2009,^36^ a lipid productivity of 53.9 mg L^−1^ day ^−1^ was observed in *Scenedesmus sp* DM. However, the aforementioned studies used an external CO_2_ supply setup (not used in the present study) for their algal cultures, which resulted in higher growth, and as such, direct comparisons cannot be made.

Our data are also in agreement with earlier findings that nitrogen sources and concentrations which result in a higher biomass yield and productivity do not produce correspondingly higher lipid content and lipid productivity. However, as per our stated hypothesis, an optimum nitrogen source and level which results in the best output as measured by critical parameters such as biomass and lipid productivity could be determined. KNO_3_ at concentrations of 2.5 - 5.0mM, followed by urea at a concentration of 5.0 mM, resulted in a comparatively good combination of biomass productivity, lipid content and lipid productivity in *S. quadricauda.* Similarly, KNO_3_ and urea at the 2.5 mM concentration outclassed all other combinations in the cases of *S. dimorphus* and *Chlorella* sp Potassium is one of the major nutrients needed for plant growth, and potassium-based fertilizers are used widely to enhance plant growth and yield in agriculture. The optimal concentration of KNO_3_ in the media for the microalgae investigated in this study was found to be 252 to 505 mg L^−1^. Potassium nitrate is also used as one of the major components in most plant tissue culture media, viz. MS media 1962 (1900 mg L^−1^),^37^ Nitschmedia 1951 (2000 mg L^−1^),^38^ Gamborg et al. media 1968 (3000 mg L^−1^)^39^ and Schenk & Hildebrandt 1972 (2500 mg L^−1^),^40^ as a source of potassium instead of KH_2_PO_4 _and KCl. Hence, potassium nitrate can be considered a dual-purpose nutrient, supplying potassium as well as nitrogen. Algal cells resemble plant cells in many aspects of their metabolism, so, obviously, higher growth was achieved when KNO_3_ was used as a nitrogen source that also met the potassium requirement of the growing cells*.* Another important outcome of this study is that better growth and productivity of the microalgae under investigation was obtained using media supplemented with 7 times less of the original nitrogen source (17.6 mM NaNO_3_) of modified BG-11 medium. Moreover, urea was also found to be an equally good nitrogen source, and it is very cheap, available in large quantities and can be exploited for large-scale cultivation. Urea utilization as a nitrogen source has also been reported in the growth of *Chlorella* sp M2.^41^

## Materials and methods

### Microalgae isolate and culture conditions

The best performing isolates *Sd12, Sq19* and *Chl16* of 3 microalgae species,* Scenedesmus dimorphus*, *Scenedesmus quadricauda* and *Chlorella* sp, were used in this study.^12,13^ Prior to inoculation, all media were autoclaved (Scimed (Asia) Pte Ltd., Singapore) at 121°C for 15 min. The inocula for different experiments were prepared by culturing algae in modified BG-11 medium (pH 7.5) by incubating (Hicon, Germany) at 18–25˚C with a 16:8 h light and dark cycle. The cultures were aerated with an air pump to avoid the settling of algae onto the surface of flasks. The cultures were grown to reach the stationary phase (18–20 days).

### Selection of suitable growth medium

In the present study, 4 different culture media, namely modified BG-11,^42^ CHU-10,^43^ BBM^44^ and F/2,^45^ were used for screening the suitable media for optimum growth. These most widely used defined synthetic media were selected for such experimental studies to avoid the confounding effects of enriched media. The chemical compositions of 4 different media are tabulated in Table 2. Nitrate and phosphate levels are exceptionally high in BG-11 medium, which is used extensively for freshwater green algae and cyanobacteria. Chu 10 was designed to match lake water, but it lacks a chelators and trace metals. Bold's Basal Medium (BBM) is another useful medium for many microalgae and contains higher concentrations of some trace elements.^46^ The experiments were done separately with each microalgae. For each medium, a set of 3 conical flasks, each containing 900 ml of medium, was inoculated with 100 ml (10% v/v) of freshly grown microalgae culture. All culture conditions were the same as mentioned above for the preparation of inocula. At the end of the experiments, when the stationary phase was reached, the algal biomass was estimated and used to extract the total lipids.

### Selection of suitable nitrogen source

This experiment was performed to study the effect of different nitrogen sources on growth and lipid production of the microalgae under investigation. Five nitrogen sources, viz. potassium nitrate (KNO_3_), urea (CH_4_N_2_O), calcium nitrate (Ca(NO_3_)_2_), ammonium chloride (NH_4_Cl) and sodium nitrate (NaNO_3_), were used in the range of 2.5 - 12.5 mM in modified BG-11 medium (by replacing sodium nitrate) while taking BG 11 medium as a control. Modified BG-11 media (900 ml) with their respective controls were inoculated with 100 ml of the respective microalgal species in one liter conical flasks. The inoculated cultures were maintained at 18–25˚C with a 16:8 h light and dark cycle by supplying aeration through an air pump. All experiments were conducted thrice and results were represented as ± s.d of 3 replicates.

### Observations and growth kinetics

In all of the experiments, the growth of the algal cultures was monitored by recording the absorbance of cultures at 730 nm using a spectrophotometer (ELICO SL-159 UV-VIS, Hyderabad, India) until the stationary phase was attained. At the same time, direct microscopic cell counts (cells ml^–1^) of the microalgal suspension cultures were also recorded using a hemocytometer. The averages of 3 replicates with 5% error bars on the 18th day of culture, when the stationary phase was reached, were plotted as a bar chart. The specific growth rate (µ) and doublings per day (K) were calculated from cell count data as described in Gour et al. 2014.^12^

### Estimation of biomass and lipid content

At the end of the experiment, when the stationary phase of the growth was reached, the cultures were centrifuged at 5000 rpm for 10 minutes (REMICPR-24, Mumbai, India) to harvest the cell biomass. Then,the cell pellets were freeze-dried and lyophilized (New Brunswick, NJ, USA). The dry weight of the microalgal biomass was determined by the gravimetrical method,^13^ and the biomass growth was represented in terms of dry weight (g L^−1^). Biomass productivity (Pdwt) was expressed as the dry biomass produced in grams per liter per day (g L^−1^ day^−1^). Lipid extraction from microalgae was done using the Bligh and Dyer method^47^ with minor modifications. Total lipid content (Lp) was calculated as a percentage of the total biomass. The lipid productivity (mg L^−1^ day^−1^) was calculated using the following formula:

Lipid productivity=biomass productivity(Pdwt)×lipid content(%)×1000/100

### Statistical data analysis

Each experiment was done in triplicate, and one-way analysis of variance (ANOVA) and the post hoc test (Tukey method) was used to test for significant differences among the treatments in the experiments.

## Conclusion

Among the 4 different media tested, the highest biomass and total lipids were recorded when microalgae was grown in modified BG-11 medium, irrespective of the microalgae species in the study. The optimal nitrogen supply, in terms of its source and a level that does not negatively affect growth but instead results in lipid accumulation in microalgae, was figured out. The highest lipid content and productivity were recorded when KNO_3_ at 2.5mM was used as the nitrogen source in modified BG 11 media. Better growth and productivity of the microalgae under investigation were obtained using KNO_3_ at a molar concentration 7 times lower than that of the original nitrogen source (17.6 mM NaNO_3_) of modified BG-11 medium.
